# Supplementary material for: An Efficient and Easy-to-Use Network-Based Integrative Method of Multi-Omics Data for Cancer Genes Discovery
Source: Front Genet. 2021 Jan 8;11:613033. doi: 10.3389/fgene.2020.613033 (PMC7820902; doi:10.3389/fgene.2020.613033)
Supplement: Supplementary Figure 1 — Comparison of precision and F1 score for different methods in TCGA-STAD and TCGA-BLCA datasets. [file Data_Sheet_1.docx]

Supplementary Material

# Supplementary Figures and Tables

## Supplementary Figures

|  |
| --- |


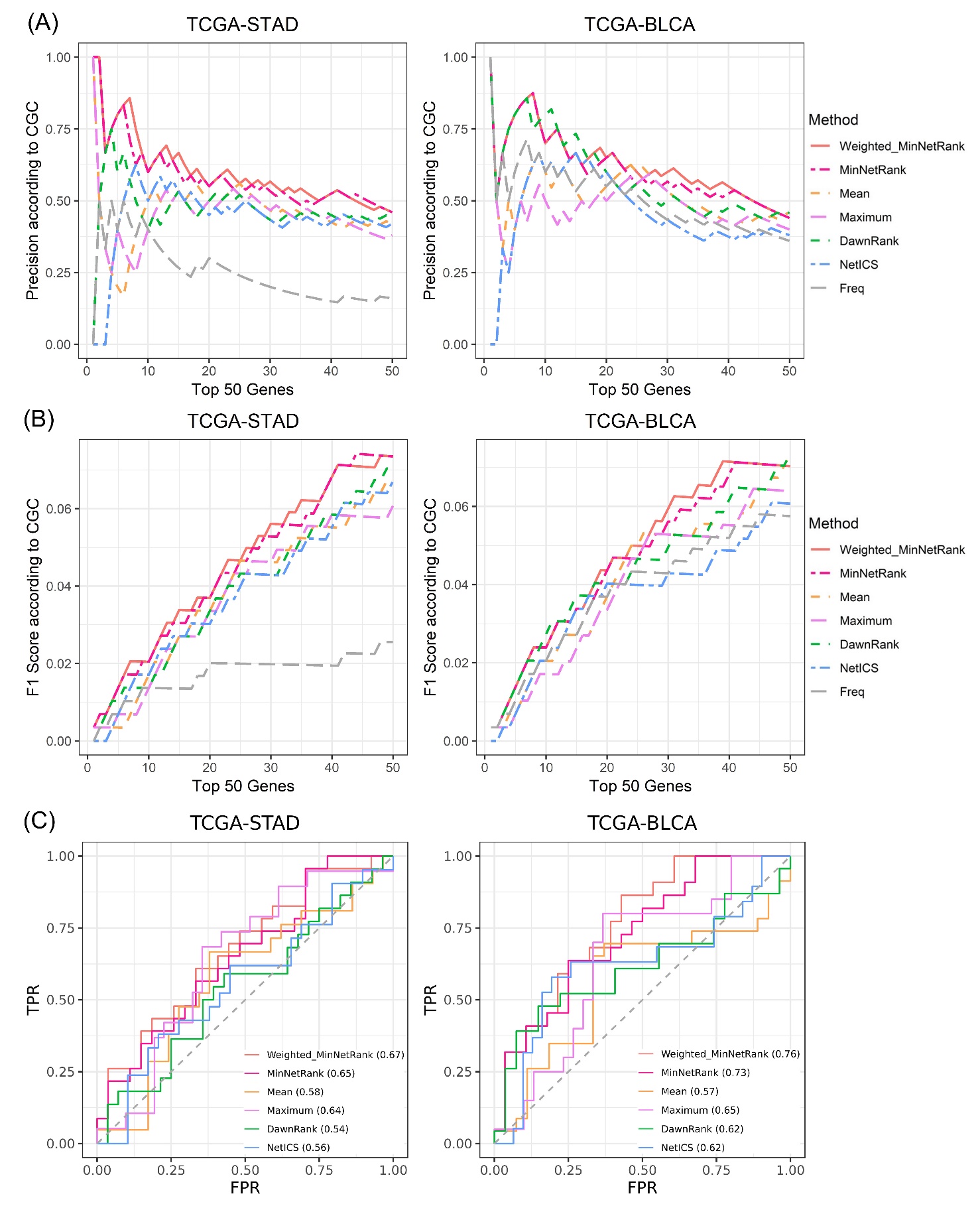


**Supplementary Figure S1. Comparison of precision and F1 score for different methods in TCGA-STAD and TCGA-BLCA datasets.** (A) The X-axis is the top 50 candidate cancer genes and the Y-axis is the precision according to known cancer genes (in CGC). (B) The X-axis is the top 50 candidate cancer genes and the Y-axis is the F1 score according to known cancer genes. (C) The ROC curve of top 50 candidate cancer genes.


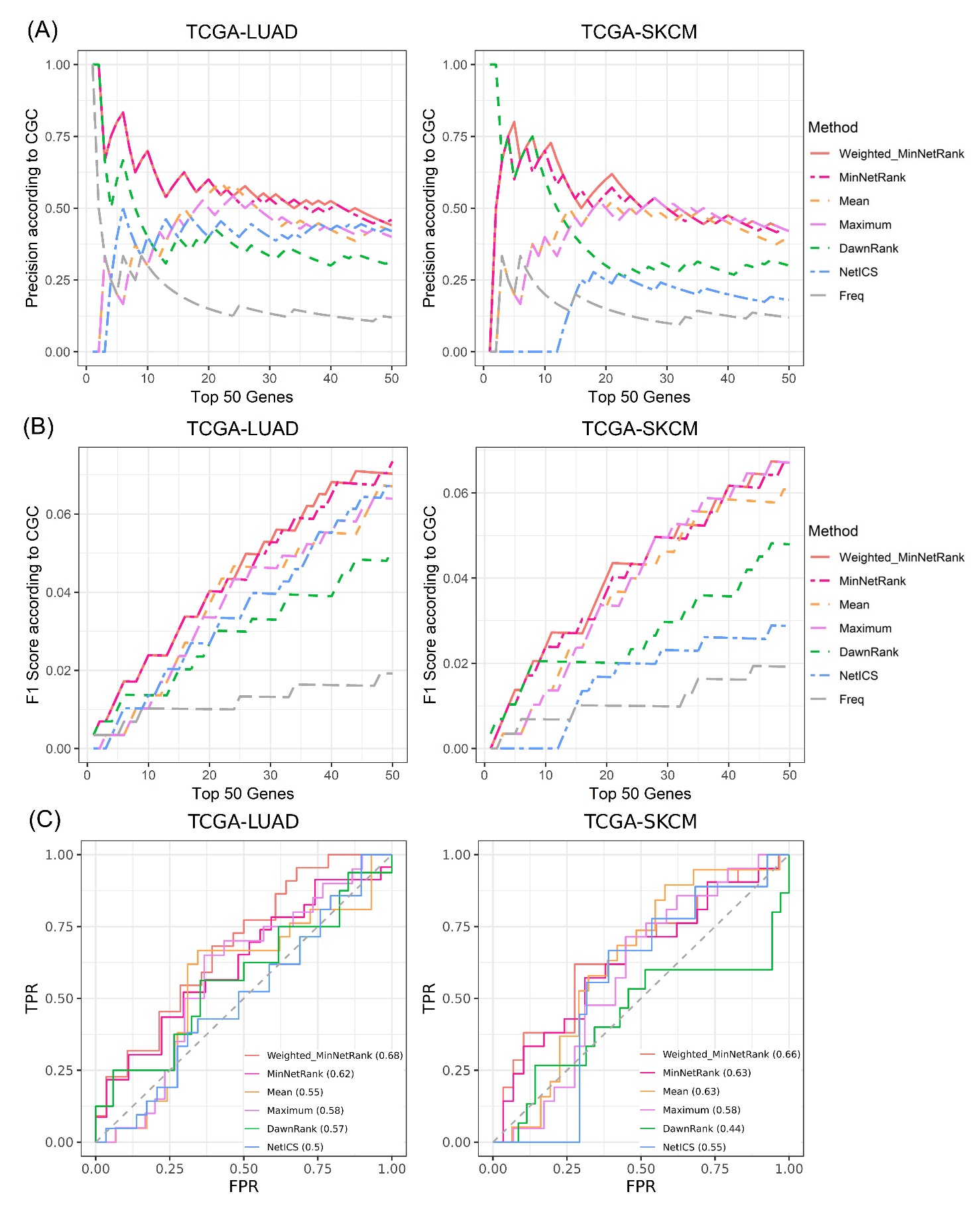


**Supplementary Figure S2. Comparison of precision and F1 score for different methods in TCGA-LUAD and TCGA-SKCM datasets.** (A) The X-axis is the top 50 candidate cancer genes and the Y-axis is the precision according to known cancer genes (in CGC). (B) The X-axis is the top 50 candidate cancer genes and the Y-axis is the F1 score according to known cancer genes. (C) The ROC curve of top 50 candidate cancer genes.

|  |
| --- |
| 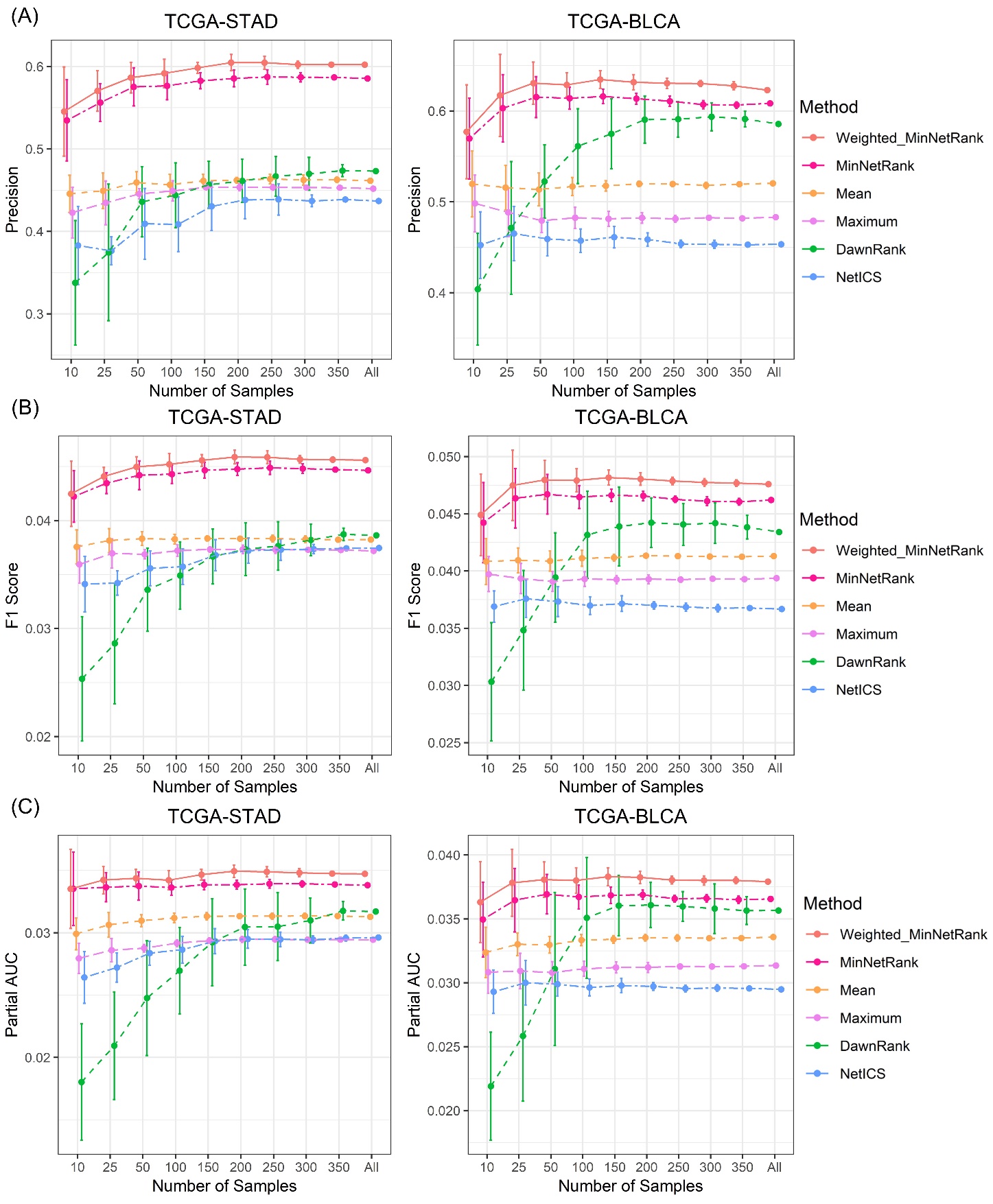 |

**Supplementary Figure S3.** **Robustness of results using the subset of samples in TCGA-STAD and TCGA-BLCA datasets.** (A) The X-axis is subset of samples. The Y-axis is mean and SD of the precision values after 10 runs using the subset of samples. (B) The X-axis is subset of samples. The Y-axis is mean and SD of the F1 Score after 10 runs using the subset of samples. (C) The X-axis is subset of samples. The Y-axis is mean and SD of the partial AUC after 10 runs using the subset of samples.

**
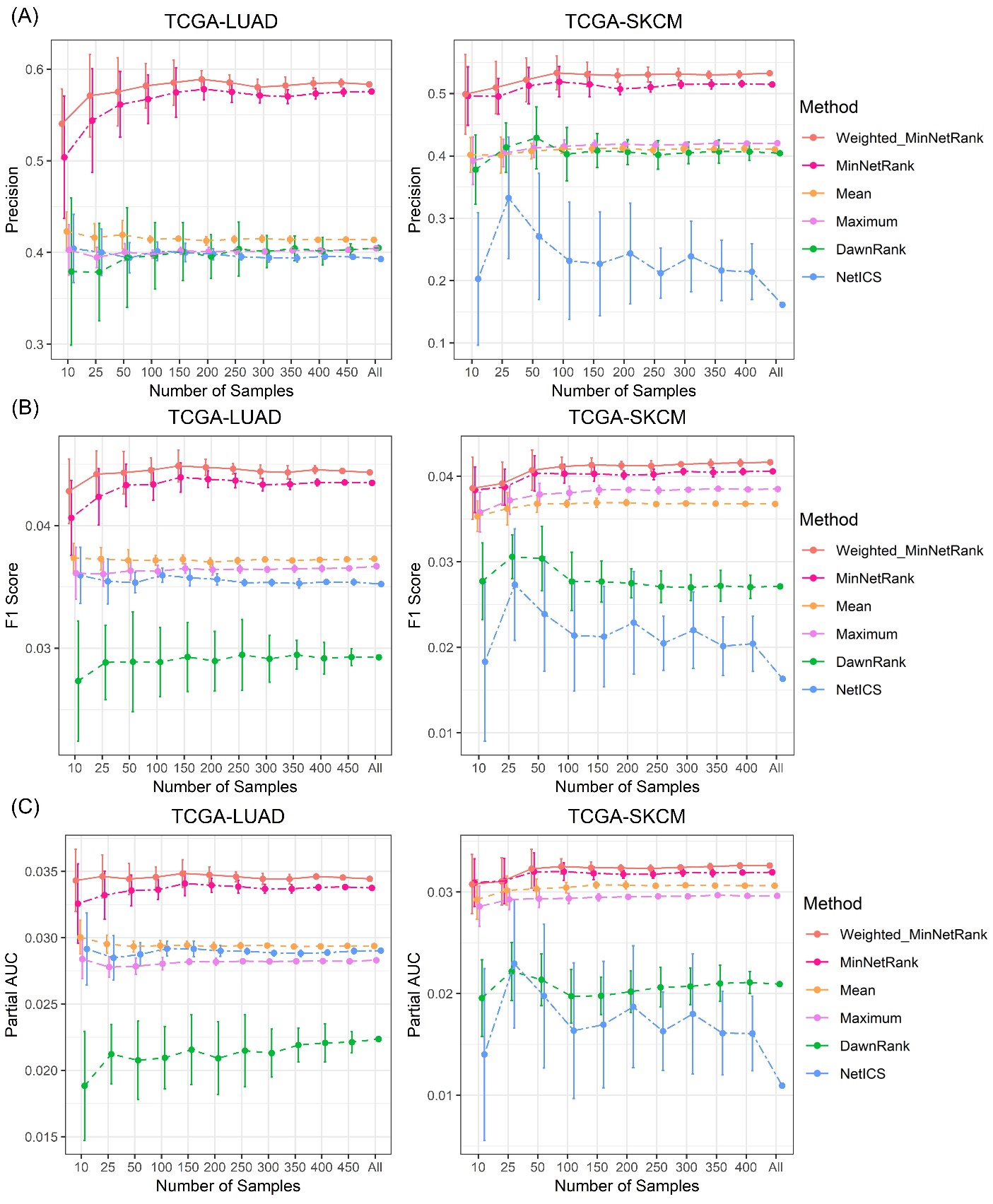
**

**Supplementary Figure S4. Robustness of results using the subset of samples in TCGA-LUAD and TCGA-SKCM datasets.** (A) The X-axis is subset of samples. The Y-axis is mean and SD of the precision values after 10 runs using the subset of samples. (B) The X-axis is subset of samples. The Y-axis is mean and SD of the F1 Score after 10 runs using the subset of samples. (C) The X-axis is subset of samples. The Y-axis is mean and SD of the partial AUC after 10 runs using the subset of samples.

| 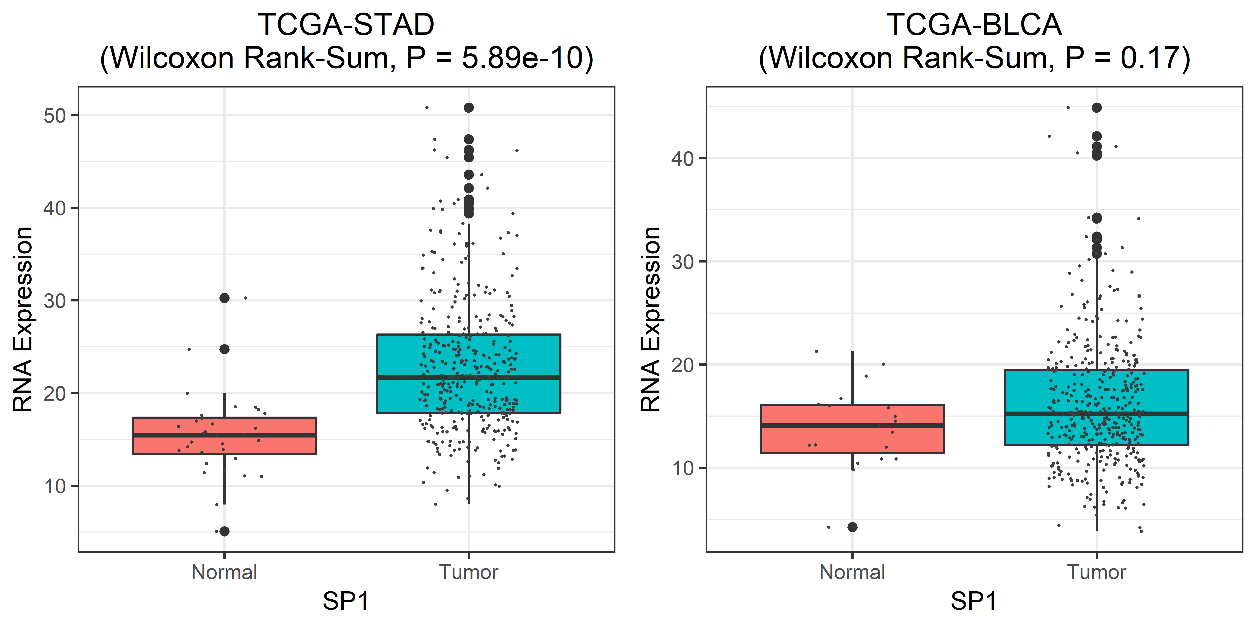 |
| --- |

**Supplementary Figure S5. The SP1 differential expression between tumor and normal in TCGA-STAD and TCGA-BLCA.**

| 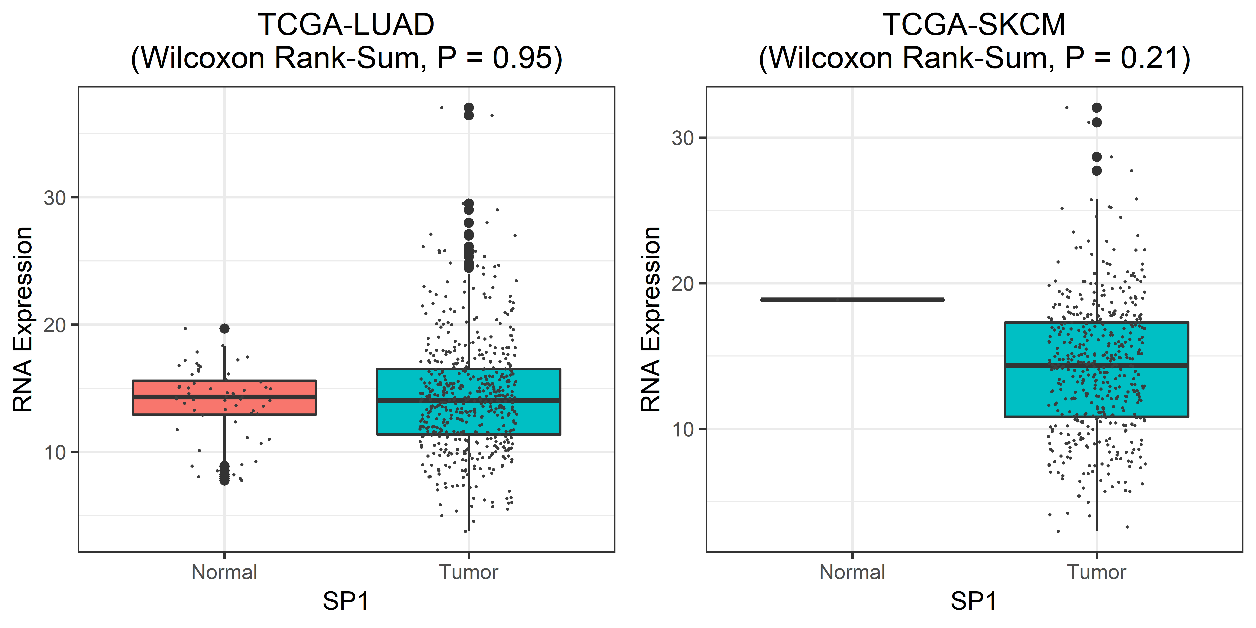 |
| --- |

**Supplementary Figure S6. The SP1 differential expression between tumor and normal in TCGA-LUAD and TCGA-SKCM.**

| 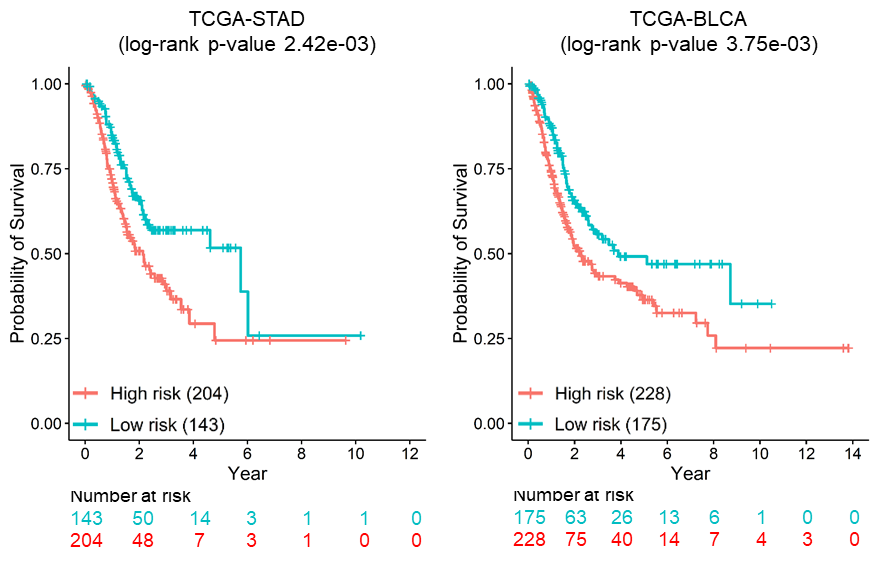 |
| --- |

**Supplementary Figure S7. The survival difference between high-risk group and low-risk group in TCGA-STAD and TCGA-BLCA.**

| 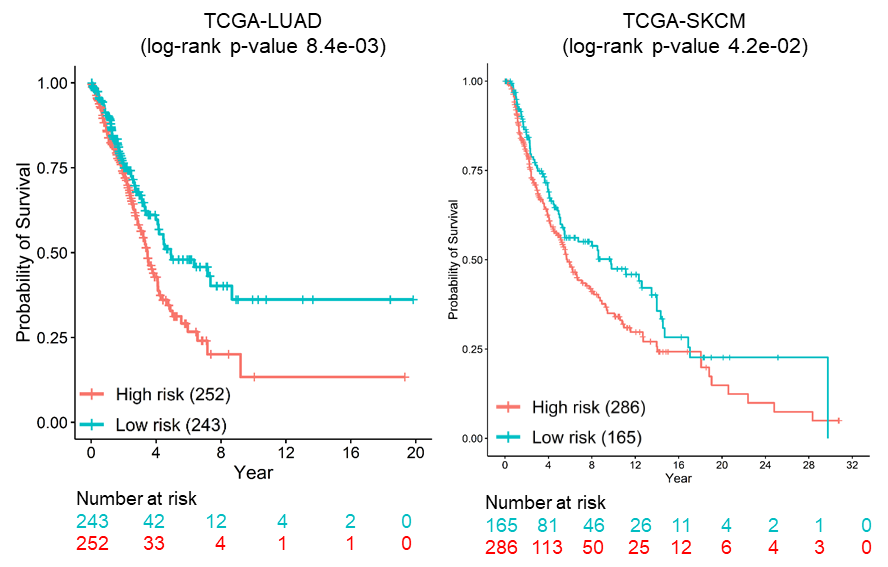 |
| --- |

**Supplementary Figure S8. The survival difference between high-risk group and low-risk group in TCGA-LUAD and TCGA-SKCM.**

## Supplementary Tables

**Supplementary Table S2. The log-rank p-value of tumor stratification for each method in six datasets**

| **Methods** | **TCGA-LIHC** | **LIRI-**  **LIHC** | **TCGA-**  **STAD** | **TCGA-**  **BLCA** | **TCGA-LUAD** | **TCGA-**  **SKCM** |
| --- | --- | --- | --- | --- | --- | --- |
| Weighted_MinNetRank | **9.21E-04** | **1.23E-05** | **2.42E-03** | **3.75E-03** | **9.21E-04** | **4.19E-02** |
| MinNetRank | **9.21E-04** | **1.23E-05** | **2.42E-03** | **3.75E-03** | **9.21E-04** | **4.19E-02** |
| DawnRank | 1.25E-05 | 2.69E-03 | 2.62E-01 | 4.18E-02 | 3.26E-01 | 4.49E-02 |
| NetICS | 7.50E-02 | 2.53E-02 | 1.20E-01 | 2.07E-03 | 1.14E-02 | 9.47E-02 |
| Mean | 1.80E-04 | 1.35E-04 | 2.65E-02 | 2.11E-02 | 1.70E-02 | 1.09E-01 |
| Maximum | 1.26E-03 | 2.10E-06 | 1.78E-01 | 6.15E-02 | 1.70E-02 | 1.09E-01 |
| Degree Centrality | 2.74E-04 | 1.18E-05 | 3.95E-02 | 1.06E-01 | 1.86E-02 | 4.85E-04 |
| Betweenness Centrality | 1.26E-03 | 2.59E-05 | 2.65E-02 | 2.11E-02 | 1.70E-02 | 4.48E-02 |
| Mean of Degree and Betweenness | 1.26E-03 | 2.59E-05 | 2.65E-02 | 2.11E-02 | 1.70E-02 | 1.09E-01 |
